# Tilapia lake virus downplays innate immune responses during early stage of infection in Nile tilapia (*Oreochromis niloticus*)

**DOI:** 10.1038/s41598-020-73781-y

**Published:** 2020-11-23

**Authors:** Kizito K. Mugimba, Mustapha Lamkhannat, Saurabh Dubey, Stephen Mutoloki, Hetron M. Munang’andu, Øystein Evensen

**Affiliations:** 1 Department of Paraclinical Sciences, Norwegian University of Life Sciences, Faculty of Veterinary Medicine, PO Box 369, 0102 Oslo, Norway; 2 Department of Biotechnical and Diagnostic Sciences, College of Veterinary Medicine Animal Resources and Biosecurity, Makerere University, P.O Box 7062, Kampala, Uganda

**Keywords:** Immunology, Microbiology

## Abstract

Tilapia lake virus (TiLV) causes high mortality and high economic losses in tilapines. We describe an experimental challenge study focusing on early post challenge innate immune responses. Nile tilapia (*Oreochromis niloticus*) were infected with 10^5^ TCID_50_/mL TiLV intraperitoneally, followed by virus quantification, histopathology and gene expression analysis in target (brain/liver) and lymphoid (spleen/headkidney) organs at 3, 7, 12, 17, and 34 days post challenge (dpc). Onset of mortality was from 21 dpc, and cumulative mortality was 38.5% by 34 dpc. Liver and kidney histopathology developed over the period 3–17 dpc, characterized by anisocytosis, anisokaryocytosis, and formation of multinucleated hepatocytes. Viral loads were highest at early time (3 dpc) in liver, spleen and kidney, declining towards 34 dpc. In brain, viral titer peaked 17 dpc. Innate sensors, TLRs 3/7 were inversely correlated with virus titer in brain and headkidney, and IFN-ß and Mx showed a similar pattern. All organs showed increased mRNA IgM expression over the course of infection. Overall, high virus titers downplay innate responses, and an increase is seen when viral titers decline. In silico modeling found that TiLV segments 4, 5 and 10 carry nucleolar localization signals. Anti-viral effects of TiLV facilitate production of virus at early stage of infection.

## Introduction

Tilapia is the second largest farmed fish species after carp globally. With such expansion there is risk of emergence of infectious diseases and tilapia lake virus (TiLV) infection has been shown to cause high mortalities in farmed tilapia over the last 4–5 years^1^. TiLV is a negative-sense, single-stranded RNA virus (-ssRNA) made of an icosahedron envelope with 55–100 nm diameter^1^. It is an orthomyxo-like virus and the only member of the genus *Tilapinevirus* in the family *Amnoonviridae*^1–3^. Since it was first reported from Israel in 2014^1^, it has been recorded in various countries globally^2,4–14^. Clinical observations include irregular swimming, skin hemorrhages, exophthalmia and ulcerative skin lesions. Histopathological liver changes are characterized as “hepatic syncytia” and thus the name ‘syncytial hepatitis’ of tilapia^1,7,15^, while brain infection has been associated with clinical symptoms of irregular swimming^1^.

Although some studies have shown occurrence of pathological changes caused by TiLV in different organs, liver and brain included, there are still few studies that describe sequential development of histopathological changes in internal organs or study host responses to TiLV infection. Moreover, sequential stages of infection progression such as virus penetration at portals of entry, and local replication followed by dispersal to target organs has not been elucidated. Establishment of infection is amongst others detected by intracellular pattern recognition receptors (mainly TLR3 and 7) leading to a cascade of cellular responses such as expression of inflammatory cytokines that modulate the migration of leukocytes to infection sites^16^. In addition, virus infected cells secrete interferons (IFNs) and IFN-inducible genes (ISGs), like Mx, are induced to limit viral replication and production of progeny^17–19^. Previously, we showed the expression of IL-1β and TNFα in response to TiLV infection in gray and red tilapia expressed at time of death^20^ but the kinetics of these genes during the incubation and acute stages of TiLV infection has not been studied. Similarly, the kinetics of PRRs, IFNs, ISGs and several other genes expressed in response to TiLV infection is not known. Further, the pathogenic sequence of events in internal organs post infection has not been studied in any detail.

The objective of the present study was to determine the progression of TiLV infection in tilapia by comparing histopathological changes in the liver (target organ), headkidney/spleen (lymphoid organ) and brain with viral loads detected at different time post of infection following an experimental challenge and to profile the mRNA expression of sensors of infection and induced innate immune (interferon-I) responses.

## Results

### Clinical signs and observations

**Figure 1:**
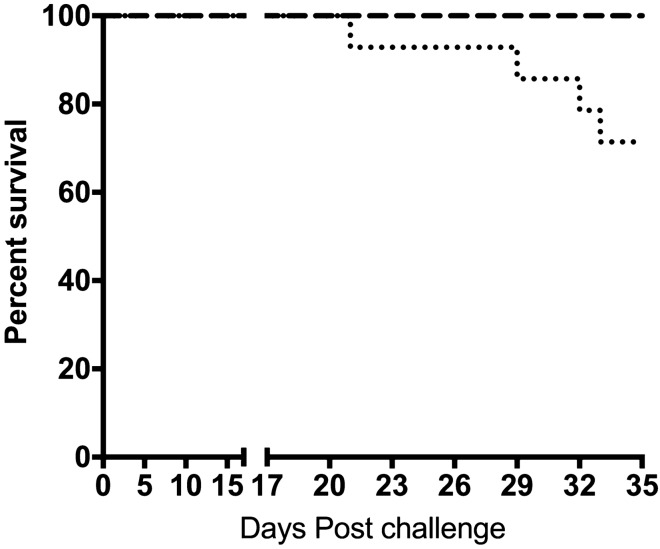
Survival curve for the challenged group (dotted line) and controls, giving a 71.5% survival (38.5% mortality) in the challenged group.

Clinical signs started as early as 3 days post challenge (dpc) characterized by loss of appetite, reduced swimming and hemorrhages on the skin surface, operculum and ventral buccal cavity. By 7 dpc there was exophthalmia in some fish, and some with abnormal swimming behavior. By 17 dpc we saw loss of balance and corkscrew swimming. First mortality was observed by day 21 and cumulative mortality was 38.5% (Fig. 1) by 34 dpc when the challenge was terminated (Fig. 1). Number of individuals at risk at day of first mortality (21 dpc) was 13 and mortality/survival was calculated on the basis of fish at risk at onset of mortality (Fig. 1).

### Viral replication kinetics

**Figure 2:**
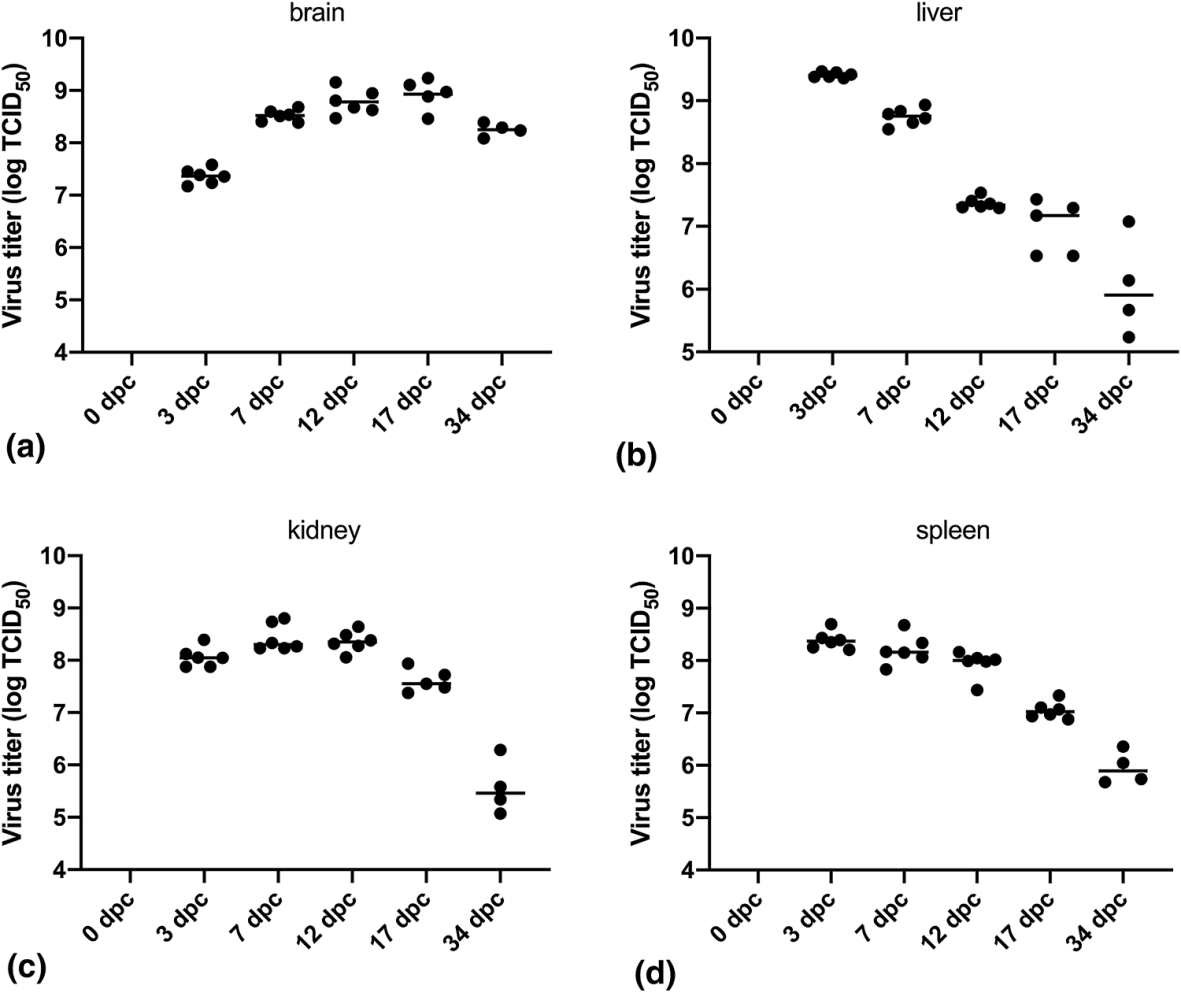
(**a**–**d**) Virus titers in the different organs shown for individual fish over the course of infection.

The viral titer in the different organs varied over the course of infection. For brain (Fig. 2a), virus titer was lowest at 3 dpc, increasing gradually up to 17 dpc (p < 0.01), and declining by day 34 dpc (compared to 17 dpc, p = 0.02), still 2–3 logs higher than other organs at end of trial (Fig. 2). Virus titer was highest in liver (Fig. 2b) and spleen (Fig. 2d) at 3 dpc with a gradual decline over the course of infection up to 34 dpc (p < 0.01). In kidney (Fig. 2c), titers were high from 3 dpc and remained high (p > 0.05) up to 17 dpc and dropped by 34 dpc (p < 0.00001). A summary for average and 95% confidence intervals for the different organs is shown in Supplementary Fig. 1.

### Histopathological changes

**Figure 3:**
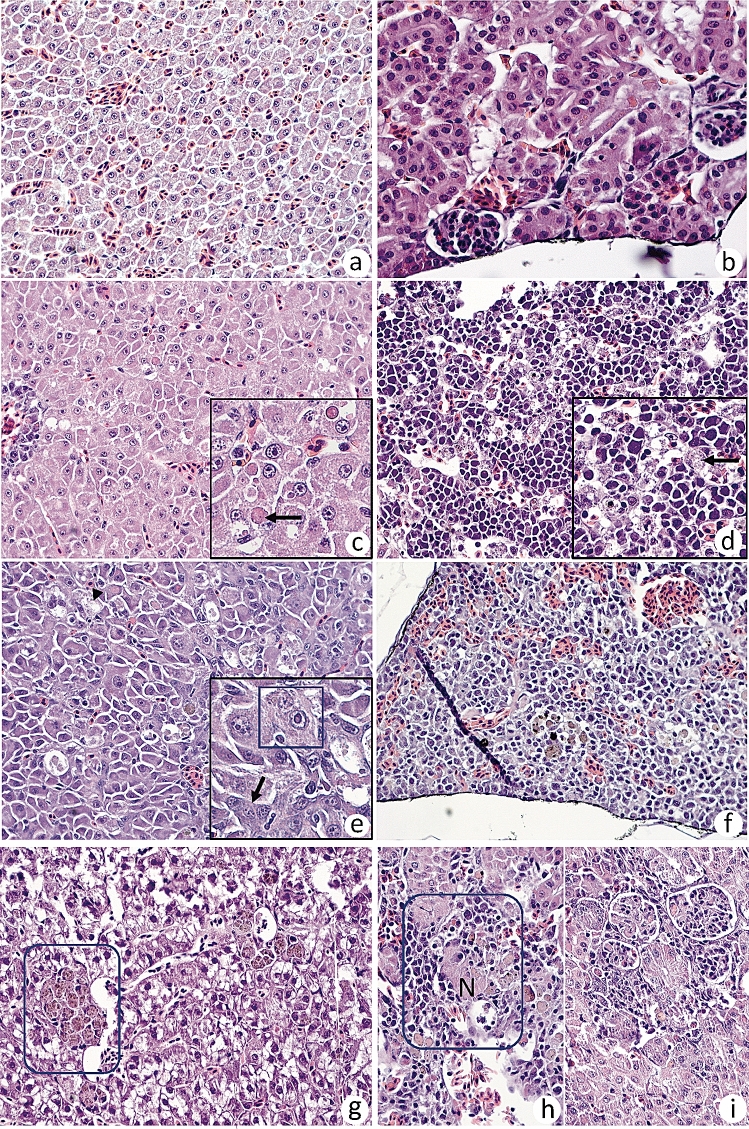

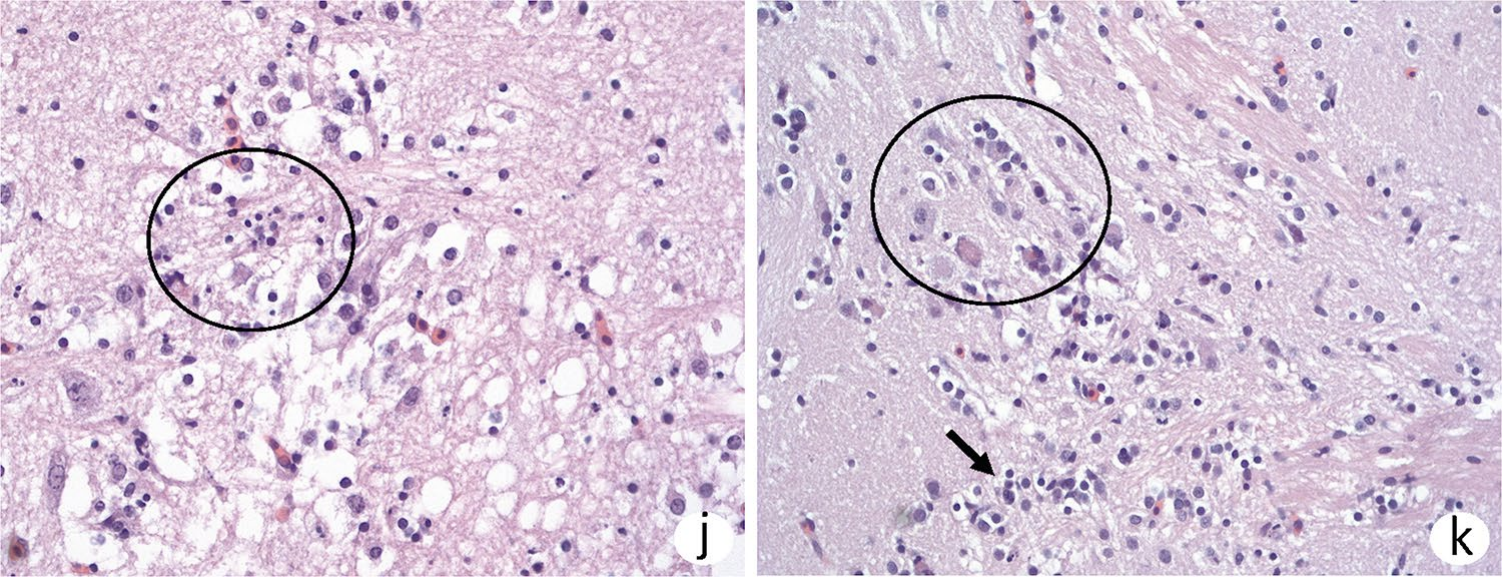
Histopathological changes in different organs over the course of infection. (**a**) Liver and (**b**) kidney prior to challenge showing mild congestion. (**c**) Liver, 3 dpc showing anisocytosis, prominent nucleoli and small droplets in hepatocyte cytoplasm (insert, arrow). Mild perivascular cuffing (arrowhead). (**d**) Kidney, 3 dpc with swollen endothelial cells containing an amorphous substance and endothelial cells fill the sinusoidal lumen (arrow, insert). (**e**) Liver, 12 dpc, with anisocytosis of hepatocytes and anisokaryosis. Prominent karyomegaly with prominent nucleoli consisting of bi- and multinucleated hepatocytes (insert). (**f**) Spleen, 12 dpc, deposition of intracellular pigment in the parenchyma. (**g**) Liver, 17 dpc, focal accumulation of brownish pigment hepatocyte cytoplasm and with vacuolated hepatocytes. (**h**) Kidney, 17 dpc, with focal, interstitial necrosis (N) and moderate infiltration of lymphocytes. (**i**) Kidney, 17 dpc, inflammatory foci around glomeruli with mild thickening of the vascular structure with deposition of amorphous substance along basement membranes. (**j**) Brain, 32 dpc, vacuoles in basal parts of the brain, pyknotic nuclei inside the circle and with macrophage-like cells. (**k**) Brain, 33 dpc, foci with increased number of inflammatory cells, also seen around arrow with increased number of inflammatory cells in white matter.

There was a mild congestion in liver and kidney in non-challenged fish at start of the experiment (0 dpc, Fig. 3a,b). Early stage liver changes post challenge (3 dpc) were characterized by degeneration and necrosis of individual hepatocytes (Fig. 3c), and accumulation of intracellular well-circumscribed, intracytoplasmic eosinophilic bodies of varying size (Fig. 3c, insert). At this time, lesions in headkidney (Fig. 3d) were characterized by swollen endothelial cells containing an amorphous substance where the endothelial cells filled almost the entire sinusoidal lumen (Fig. 3d, insert). At later stage, 12 dpc, changes in the liver were characterized by anisocytosis of hepatocytes and anisokaryosis (Fig. 3e). Hepatocytes also showed karyomegaly with prominent nucleoli, and bi- and multinucleated hepatocytes (Fig. 3e, insert). Mild congestion was seen in the spleen at this time and with deposition of pigment in the parenchyma (Fig. 3f). By 17 dpc, liver changes were characterized by focal accumulation of brownish pigment in the cytoplasm of hepatocytes, and also vacuolated hepatocytes (Fig. 3g). Kidney changes at this time were observed as focal, interstitial necrosis and moderate infiltration of lymphocytic inflammatory cells (Fig. 3h). Similar inflammatory foci were found around glomeruli with mild thickening of the vascular structure of the glomeruli with deposition of an amorphous substance (Fig. 3i). Brain changes were not observed prior to 30 days post challenge, and only 2 of 6 fish examined at 30 days post challenge). Changes by 32 dpc were characterized by vacuole formation in basal parts of the brain with pyknotic nuclei and presence of macrophage-like cells (Fig. 3j). Foci with infiltration of inflammatory cells were found in white matter (Fig. 3k, 33 dpc).

### Gene expression analysis

#### Expression of toll-like receptors 3 and 7 mRNA

**Figure 4:**
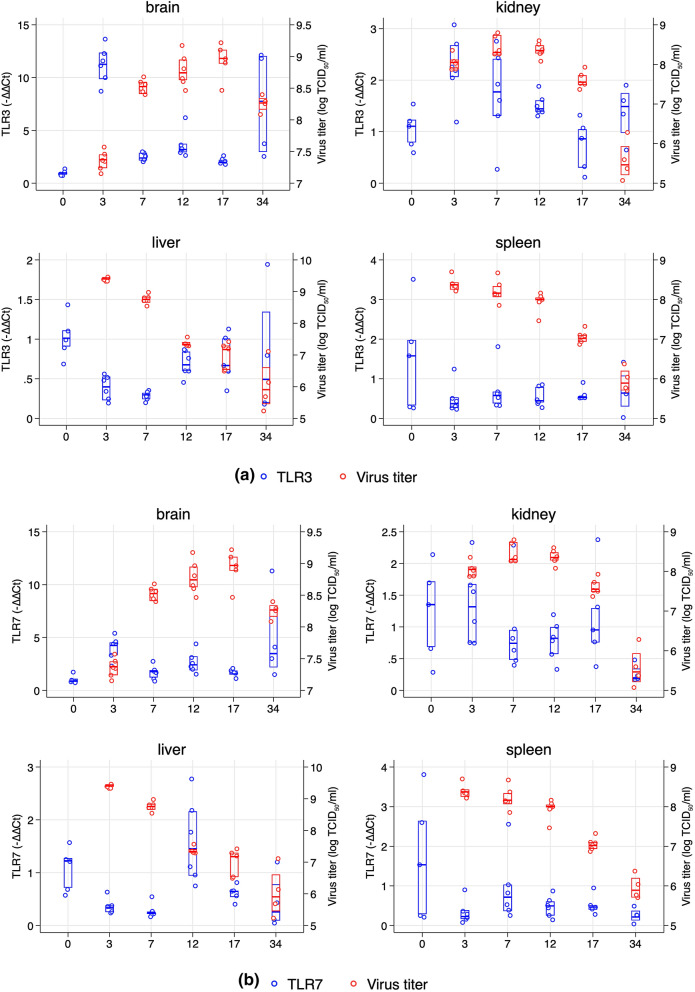
(**a, b**) TLR3 (**a**) and TLR7 (**b**) expression in the different organs over the course of infection plotted against viral titers in corresponding organs. X-axis shows days post challenge while the y-axis shows relative gene expression (2^ddCt^-left axis) and virus titer (log TCID_50_, right axis). Median and 25/75% percentile is shown for TLR3/7 and virus titer.

TLR3 and TLR7 mRNA expression was profiled in the different organs over the challenge period and expressed with virus titer at the same time points. TLR3 was found upregulated in brain and kidney at early time post challenge, 3 dpc when virus titers were low (Fig. 4a). For liver and spleen there was a down-regulation at 3 dpc (relative to day 0) with increasing virus titer for TLR3/7, marked in liver and moderate in spleen (Fig. 4a). At later time, liver showed a moderate increase for TLR3 as virus titers declined, while spleen TLR3 expression remained low with a weak increasing trend towards 34 dpc (Fig. 4a). For TLR7, expression declined with increasing virus titer in all organs at early time post infection as virus titers peaked (Fig. 4b).

**Figure 5:**
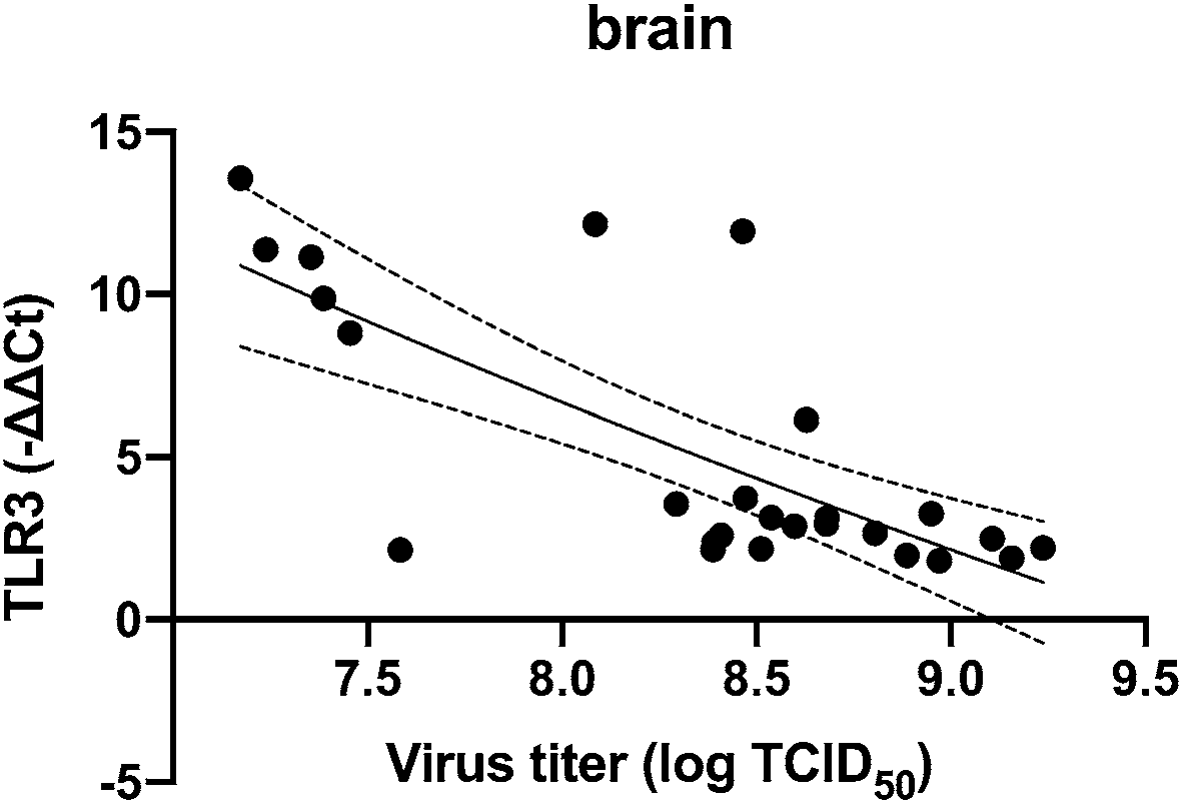
TLR3 expression plotted against virus titer for brain for all time points post challenge. Notably, as virus titers increase (along x-axis), TLR3 expression drops (dpc 7–17). Correlation between virus titer and TLR3 expression in brain for all time points post challenge, dotted line indicated 95% CI.

Plotting TLR3 expression against virus titers for brain over the course of challenge, we found a significant and inverse relationship between viral loads and TLR3 expression (Fig. 5), r^2^ = − 0.8 (p < 0.0000).

#### Expression of IFNβ and Mx genes

**Figure 6:**
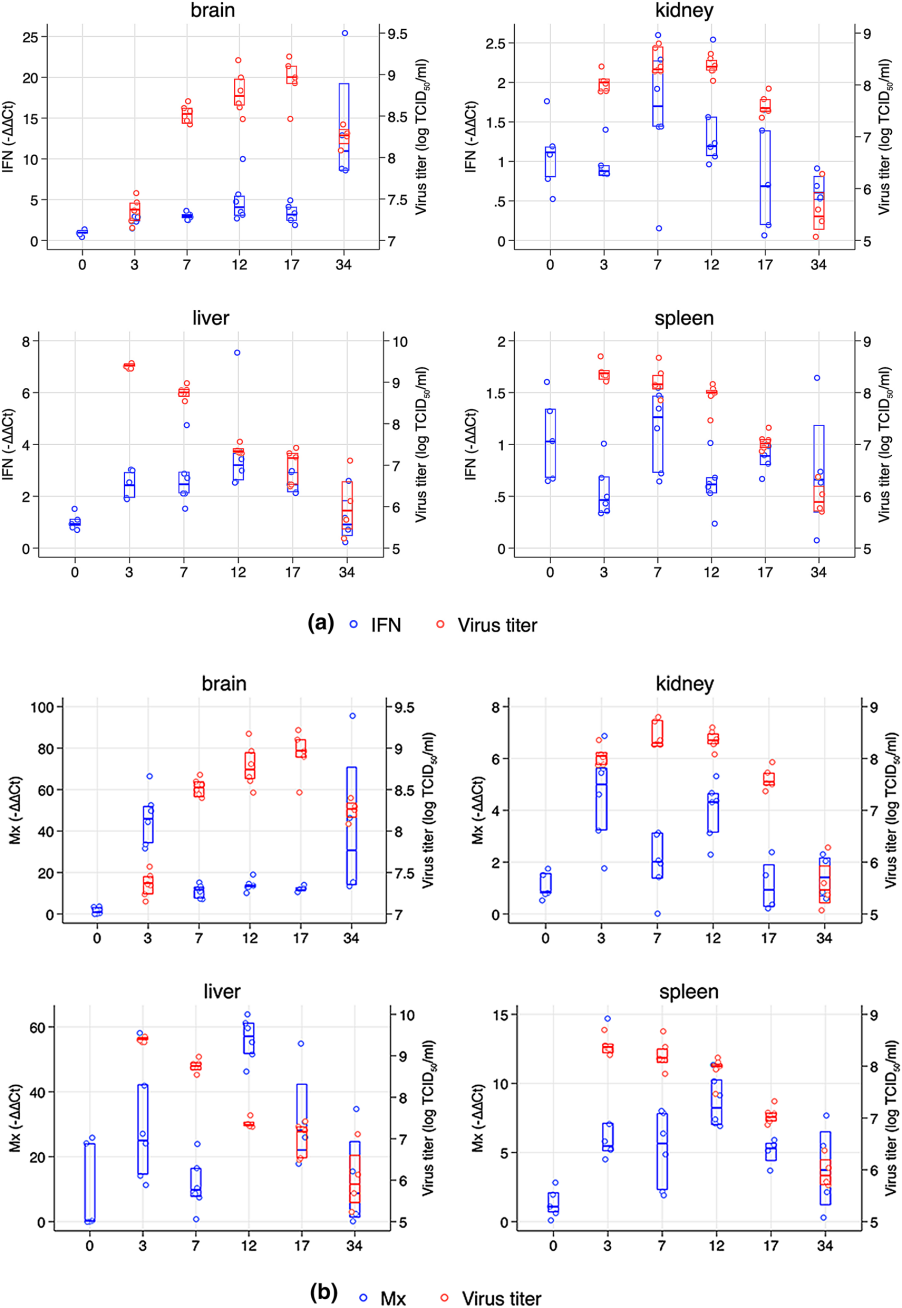
(**a**) IFN-ß expression in all organs over the course of the experiment. X-axis shows days post challenge while the y-axis shows relative gene expression (2^ddCt^- left axis) and virus titer (log TCID_50_, right axis). Median and 25/75% percentile is shown for IFN and virus titer. (**b**) Mx expression and virus titer in different organs over the course of the experiment. X-axis shows days post challenge while the y-axis shows relative gene expression (2^ddCt^-left axis) and virus titer (log TCID_50_, right axis). Median and 25/75% percentile is shown for Mx and virus titer.

IFN-β and Mx levels were low at time 0 (pre-challenge). In brain there was no significant upregulation of IFN-ß by 3 and 7 dpc (p > 0.05) although virus titers were as high as 3.8 × 10^7^ TCID_50_ by 3 dpc and 3.4 × 10^8^ TCID_50_ by 7 dpc (Figs. 2a and 6a). By 12 dpc there was a significantly increased expression of IFN-ß in brain (p = 0.003, compared to time 0) and virus titer had increased further to 3.162 × 10^8^ TCID_50_ (log_10_ 8.5) at this time (Figs. 2a, 6a). IFN-ß levels increased by 34 dpc (compared to 12 and 17 dpc) and by this time virus titer had dropped by 0.7 log_10_ (from 17 to 34 dpc, Figs. 2 and 6).

Mx expression in brain was upregulated at 3 dpc (p < 0.0001) but in general inversely correlated with virus titers (r^2^ = − 0.66, p = 0.0003, Fig. 6b, Supplementary Fig. 2). IFN-ß and Mx expression in brain correlated, r^2^ = 0.64, p = 0.0001) over the course of the experiment (Supplementary Fig. 3).

In kidney, IFN-ß expression was not upregulated at any time compared to pre-challenge levels, but highest expression was at 7 dpc (p = 0.07; 7 dpc compared to 3 dpc). By 12 dpc, IFN-ß was reduced (non-significantly compared to 7 dpc) while virus titers remained high over this period (log 8.4 TCID_50_ at both time points). Later, IFN-ß expression declined with declining virus titer (Fig. 6a). Mx expression was significantly upregulated at 3 dpc (p = 0.0003), and dropped by 7 dpc (p = 0.025) as virus titers increased significantly from 3 to 7 days (p = 0.0247, Fig. 6b). Later virus titers dropped as did Mx expression (Fig. 6b).

In liver, IFN-ß increased from day 0 to 3 dpc (expression remained low at peak virus titer, 3 and 7 dpc (peaked at 12 dpc (p = 0.0013 compared to day 0) (Fig. 6a) which is after virus titers had dropped by 2 log_10_ from 3 dpc (Figs. 2c and 6a). IFN-ß was not differently regulated at any other time points post challenge, apart from 34 dpc when it fell to pre-challenge levels. Mx expression reached its highest level at 12 dpc when virus titers had dropped from its peak at 3 dpc (Fig. 6b).

In spleen, IFN-ß expression was not upregulated at any time post challenge (compared to pre-challenge levels, p > 0.05, Fig. 6a) despite virus titers were above 8 log_10_ TCID_50_. There was no correlation between virus titer and IFN-ß expression over the challenge period (p = 0.12). Mx expression showed a significant upregulation from 0 to 7 dpc (p = 0.006) but did not increase any further up to 12 dpc (p > 0.11) when virus titers were high (> 8 log_10_ TCID_50_).

### IgM expression

**Figure 7:**
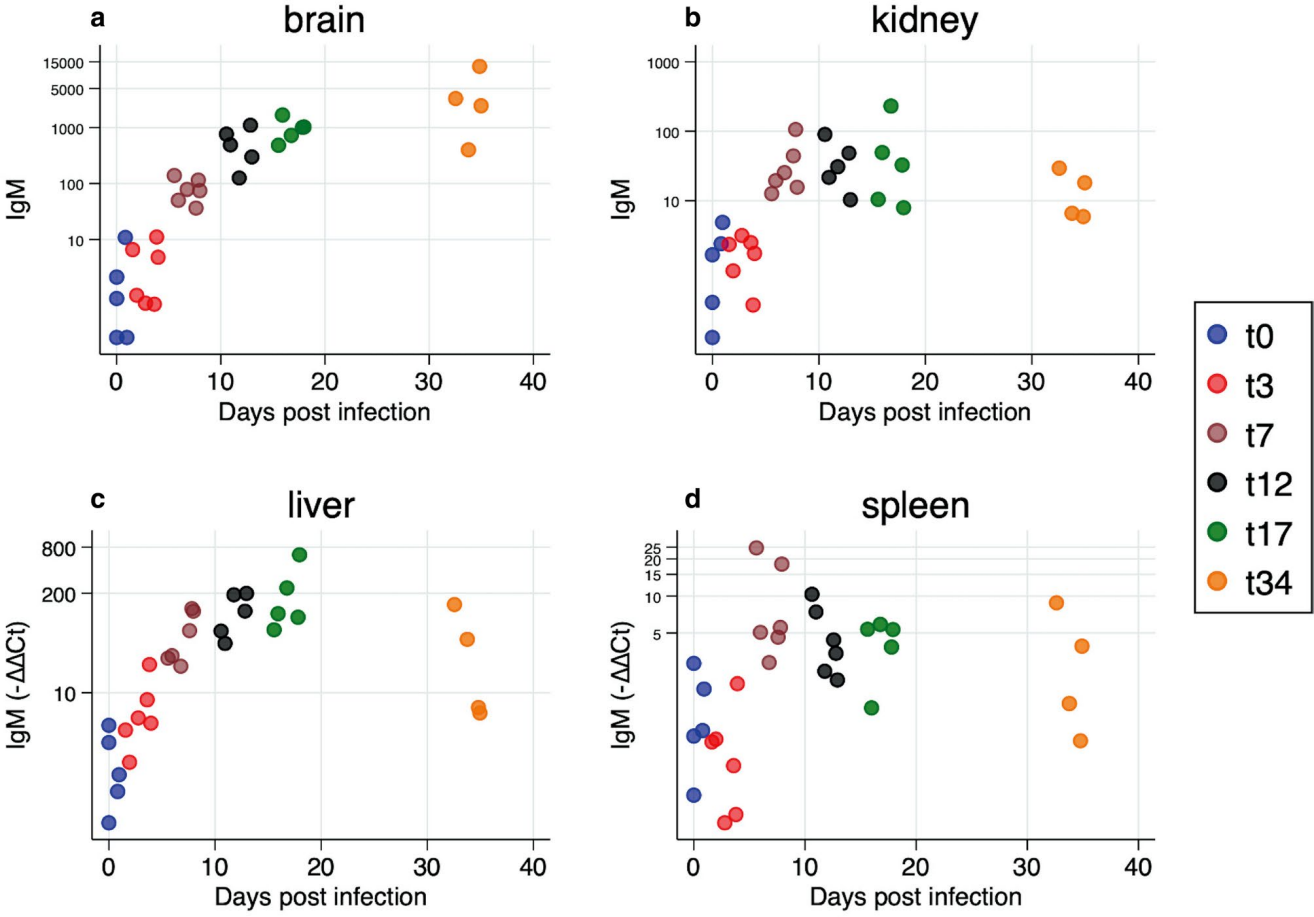
IgM mRNA expression in (**a**) brain, (**b**) kidney, (**c**) liver and (**d**) spleen over the course of infection showing a gradual increase from day 7 and onwards for all organs. There is similar pattern in brain and liver, while kidney and spleen are more variable, but an increase is seen during the first part of the challenge. Brain is highest at 34 dpc, different from all other organs.

All organs had low levels of IgM mRNA expression at time of challenge and at 3 dpc (p > 0.05). From 7 dpc, there was a significant increase in IgM mRNA levels in brain (p = 0.0002, Fig. 7a) and there was a good correlation between time post challenge and IgM mRNA expression (r^2^ = 0.67). Headkidney (Fig. 7b) and liver (Fig. 7c) show a similar pattern from 3 to 17 dpc, although with lesser upregulation than brain, and there was a numerical but not significant decline (p = 0.22 for both) by 34dpc. Spleen (Fig. 7d) show lesser upregulation over the course of challenge, although significant by 7 dpc (p = 0.006) and a decline (not significant) by 12, 17 and 34 dpc.

### In silico analysis of TiLV genome

On the basis of infected fish showing distinct nuclear and nucleolar changes morphologically, we analyzed all segments of TiLV for presence of nuclear and nucleolar localization signals, and also for motifs implicated in down-regulation of anti-viral responses, motivated from the observation that infection at early stage showed down-regulation of innate responses. Here we found nucleolar localization (NoLS) in segments 4 (KVLRASEKKRERENAKKSRKAPEA, positions 85-108), 5 (WCFKTFFYIKKRLKKKSPLEDDEVPL, positions 313-339) and 10 (GCLVLRSRKIKKGKKAASKKRSWKNERYGAD, positions 19-49). And for segment 10, nuclear localization signals were also detected (RVDFNPKNRRDREDDGQSDLSRFSEDFGKKSLD, positions 78-110).

## Discussion

The main conclusion that can be drawn from this study is that sensors (TLR3/TLR7), mediators (IFN-ß) and effectors (Mx) of innate responses are downplayed in several target organs at early time of TiLV infection in tilapia. Downplaying the innate responses could explain why virus titers peak at early stages of infection, ultimately resulting in fish mortality. The mortality rate in the current study was 38.5% compared to that reported previously^4,21^. This could be due to the different strains of virus and different tilapia strains used in the studies. More studies are needed to confirm if there are species differences in susceptibility to TiLV which would be of interest for breeding for resistance. The primary replication of virus takes place in the liver, kidney and spleen and later proliferates in the brain where the infection is maintained over time. Sensing of virus infection (TLR3/7) and IFN-ß expression shows no upregulation in response to virus infection at early time, 3 and 7 dpc, post challenge in brain, liver, spleen or kidney, despite virus titers being high at these time points. IgM expression increases over the course of the experiment in all organs tested.

Orthomyxoviruses have a number of mechanisms that counteract the IFN α/ß-system, which is accomplished by inhibiting activation of key cellular proteins by virus-encoded proteins^22^. Infectious salmon anemia virus (ISAV), a pathogenic orthomyxovirus of fish, has been found to delay induction of type-I interferon responses in infected cells of salmonid origin^23^. Such mechanisms are not studied at all for TiLV infection in vitro or in vivo*,* and the fact that so little is known about that the function of the different proteins encoded by TiLV makes such studies challenging and time consuming. That said, there is a consistent observation in this study that early sensors of virus infection (TLR3/7) are downplayed at early time of infection as are IFN-ß and Mx responses, concomitant with high virus titers. This provides circumstantial evidence that TiLV exploits mechanisms similar to viral species in the family *Orthomyxoviridae.* These findings warrant further investigations to better understand the host-virus interplay of TiLV and susceptible host species or permissive cell lines. TLRs 3 and 7 are intracellular PRRs localized in endosomes^24^. TLR7 only recognizes viral ssRNA^25,26^. Activation of TLR3 receptor by dsRNA activate the NF-kB pathway, which leads to production of type I IFNs. As such, several studies have shown positive correlation between TLR3 and type I IFN expression^27,28^. Similarly, we found a positive correlation between TLR3 and IFNβ as well as between TLR3 and Mx expression in the brain. Hence, it is likely that TLR3 had a dual function serving as a dsRNA sensor for TiLV as well as an antiviral compound limiting TiLV replication. However, there is need for further investigation to demonstrate the activation of IFNs by TLR3 in TiLV infected cells.

The histopathological changes observed in internal organs, liver and kidney, are typical of TiLV infection^7,29^. The most typical finding is the formation of multinucleate cells (hepatocytes), referred to as syncytia in previous publications^7^. The question is if previous observations are indicative of true syncytia or nuclear division without concurrent cytoplasmic separation, and this remains to be shown. Several viral species are known to produce proteins that can result in cell fusion, and membrane fusion is a crucial step when viruses enter their target cells typical for influenza^30^ and HIV-1^31^. More studies are required to decide what underlying mechanisms are at play and particularly if the observed changes represent true syncytia or not. Hepatocytes of infected fish showed distinct anisocytosis and nucleoli of various forms and sizes (anisomorphic, Fig. 3e). Several viral species are known to locate different viral proteins in the nucleoli with the aim to promote production of viral proteins that again promote virus replication^32^. This is interesting in light of the finding that sensors and effectors of viral infection are downplayed at early stage of experimental infection but here, additional studies are required to understand the detailed mechanisms involved.

Manifestation of clinical nervous signs characterized by the corkscrew swimming behavior progressively was seen by 3 dpc and more prominent at 17 dpc, which coincided with the progressive increase in viral loads in the brain whose peak was at 17 dpc. Histopathological changes in the brain were observed from 30 dpc and consisted of vacuole formation in basal parts of the brain with pyknotic nuclei and presence of macrophage-like cells and small inflammatory foci were found in white matter. The viral load in the brain increased at a later stage than in internal organs and while there is indication of brain being a secondary replication site for TiLV, this cannot be fully confirmed since the fish were followed for only 5 weeks post challenge. A study of subclinical infection in Nile tilapia showed that virus genome was not found in brain in contrast to spleen and kidney^12^. So, from the current study it is not possible to define the exact sequence of events for primary and secondary replication sites for the virus. Viral loads are high at early time of infection in the headkidney and spleen but to what extent this would be typical of infection through natural routes remains to be shown. Challenge in this study was carried out by intraperitoneal injection and this method was used to ensure that all fish received the same virus dose. Comparing early stage responses using immersion or bath challenge where the dose given to individual fish would have been difficult to assess would have made it even more difficult to interpret immune responses. Our thinking is that drainage of virus from site of injection would include headkidney and spleen at early stage and these lymphoid organs served as primary replication sites, although this remains to be shown. Similarly, virus is taken up in the liver at early stage which also serves as an amplification site before the virus enters the brain. Other studies have shown that the infection is reproduced through intra-gastric exposure^33^ and in early studies it was shown that TiLV-infected fish also exhibit changes in the gastro-intestinal tract^7^. To better understand the progression of TiLV infection in different organs, there is need to compare infection induced by i.p. with infection established through natural routes such as the gills and skin.

Compared to other viral species with an ability to infect the brain, Poisa-Beiro et al.^34^ found excessively high Mx levels in the brain (target organs), much higher than in the headkidney (lymphoid organ) in sea bream infected with nervous necrosis virus (NNV). Similarly, we found higher IFNβ and Mx levels in target organs (brain and liver) than in lymphoid organs (spleen and headkidney) and Mx expression was significantly higher in brain at all time points post challenge compared to spleen and kidney (Supplementary Fig. 4). It would be interesting to explore in more detail to what extent high Mx and IFN-ß levels expressed in the brain and liver could serve as molecular markers of TiLV infection and also to what extent it impacts outcome of infection.

Up-regulation of IgM mRNA in different organs post infection has been reported in different fish species like sea bass (*Dicentrarchus labrax*)^35^, turbot (*Scophthalmus maximus*)^36^, and rainbow trout (*Oncorhynchus mykiss*)^37^. In tilapia, Yin et al.^38^ showed IgM expression in various organs such as headkidney, spleen, thymus, liver, muscle, intestine, gill and skin post *S. agalactiae* infection, as early as 12 h post infection to highest levels by 5 dpc in the intestine and headkidney. We find that IgM levels were higher in target organs (liver and brain) than in lymphoid organs (headkidney and spleen). We observed a gradual increase in IgM mRNA expression from significantly low levels at 3 dpc to high levels reaching the peak at 12 dpc in the liver and headkidney. This was followed by a sharp decline which could be associated with IgM being used in virus neutralization (antibody consumption) contributing to reduction of viral loads at later timepoints as observed for other virus infections in fish^39^. In the brain, IgM showed an exponential increase that corresponded with increase in viral loads. Increase in IgM mRNA expression likely reflected in increased local production of IgM, could explain the decline in viral load. This remains a hypothesis since there are no studies addressing the importance of antibodies in protection against infection/disease.

We describe in this study the interplay between TiLV and innate immune responses of experimentally infected tilapia showing that high virus titers without concomitant upregulation of innate sensors and effectors. There is a need for probing deeper into the underlying molecular mechanism mediating interactions and responses, with a particular focus on possible nuclear and nucleolar localization of specific virus proteins and how this impact immune responses.

## Materials and methods

### Cell culture and virus preparation

Tilapia fin cells 10 (TFC#10)^12^ were cultured in L-15 medium (Leibovitz) supplemented with Glutamax (Gibco, Carlsbad, CA, USA), 20% fetal bovine serum (FBS) (Sigma Aldrich, St. Louis, MO, USA) and 1% gentamycin in 162 cm^3^ flask at 28 °C. When the flasks were 80% confluent, cells were infected with TiLV (KU552132) at a multiplicity of infection (MOI) 1:10 followed by incubation at 28 °C and daily observation under a light microscope for cytopathic effect (CPE). When total CPE was observed, the supernatant was harvested by centrifugation at 2500 rpm for 10 min followed by filtration using Whatman 45 µm filters (GE Healthcare Life Sciences, Norway) to remove all cell debris. The concentration of virus in the supernatant was determined using the tissue culture infective dose 50 (TCID_50_/mL) method^40^.

### Challenge model experiments in fish

This experiment was performed in the wet laboratory at the Faculty of Veterinary Medicine, Norwegian University of Life Sciences. A total of 78 healthy Nile tilapia (*Oreochromis niloticus*) weighing approximately 30 g each were used in the study. Fish were kept in stagnant, dechlorinated, purified freshwater, at a temperature of 28 °C, in 50 L, transparent aquaria, where 30% of water volume was replaced every day. Six fish were randomly selected and used for screening of TiLV and bacterial infections prior to onset of the experiment, and all fish were found non-infected. Water was filtered before use (0.22 μm filter) and no other fish were kept in the same unit during the experiment to ensure no horizontal transfer of infection from other fish. The remaining 72 fish were randomly put into four tanks each containing 18 fish. A total of 36 fish in Tanks 1 and 2 (challenge tanks) were injected intraperitoneally with 10^4^ TCID_50_/fish TiLV (0.1 ml/fish at a concentration of 10^5^ TCID_50_/ml). Another 36 fish were transferred to tanks 3 (18 fish) and 4 (18 fish) and these were injected with 0.1 mL phosphate buffered saline (PBS). All fish were anaesthetized using benzocaine prior to challenge. The animal experiment was approved by the unit’s animal ethics committee (Institutional Animal Care and Use Committee/IACUC) and the Food Safety Authority and executed in compliance with the local and national regulations associated with laboratory animal experiments. All fish were fed ad libitum using commercial feed (Skretting, Norway). Prior to challenge (t = 0), 5 fish were collected as reference at start (tanks 3 and 4). At 3, 7, 12, and 17- days post challenge (dpc) fish were sampled from challenge tanks, n = 6, 6, 6, and 5 fish per time point (equal numbers per tank; 3 and 2 fish at 17 dpc), and at 34 dpc, 4 fish were collected (2 from each tank). The same number of control fish (PBS group) were collected per time point as for the challenged fish. At each sampling time brain, liver, headkidney and spleen samples were collected and stored in RNA*later* for virus quantification and assessment of gene expression. For histopathology liver, kidney, spleen and brain samples were collected and submerged in 10% phosphate-buffered formalin.

### RNA extraction and cDNA synthesis

Total RNA was extracted using a combination of the TRIzol (Gibco, Life Technologies) and RNeasy Mini kit (Qiagen, Hilden, Germany) techniques as previously described^41^. Briefly, 30 mg of tissue was homogenized in 1 mL TRIzol followed by spinning at 12,000 *g* for 10 min at 4 °C. Thereafter, the supernatant from each Eppendorf tube was transferred into a new tube to which 0.2 mL chloroform was added. This was followed by vortexing for 15 s and incubation at room temperature for 5 min. After spinning at 12,000 *g* for 15 min, the aqueous phase was transferred into a new tube to which 600 µL of 70% ethanol was added. After vortexing, the contents were transferred to RNeasy spin columns. Thereafter, the Qiagen protocol was used following the manufacturer's protocol (Qiagen, Hilden, Germany). In the final step, total RNA was eluted in RNAse free water. Quality assessment and quantification was done using a spectrophotometer (NanoDrop ND‐1000, Thermo Scientific Inc).

The synthesis of cDNA was done in 20 μl reaction volumes using the transcriptor first strand cDNA synthesis kit (Roche Diagnostics) having an integrated step for contaminated genomic DNA removal (Roche Diagnostics). In the first step, a total volume of 13µL constituted by mixing 1 µg of template RNA with 2.5 µM Anchored-oligo (dT)_18_ primer and 60 µM random hexamer was used for denaturation by heating at 65 °C for 10 min to remove secondary RNA structures followed by cooling at 4 °C. Thereafter, 8 nM MgCl_2_, 20U of Protector RNase inhibitor, 1 mM dNTPs and 10U of transcriptor reverse transcriptase was added to each tube to make a final volume of 20 µL. In the second step, all tubes were subjected to heating at 25 °C for 10 min, 50 °C for 60 min and final reverse transcriptase inactivation at 85 °C for 5 min. The reverse transcriptase used has RNase H activity that removes RNA remnants in order to improve the downstream cDNA applications. Synthesized cDNA was stored at − 80 °C until use.

### Gene expression analysis and virus quantification

Gene expression analysis was done using quantitative real-time PCR (qRT-PCR) using the SYBR green kit (Roche Applied Science) using cDNA from liver, brain, headkidney and spleen samples. Primers used were designed using the CLC Workbench version 6 and sequences are shown in Table 1. qRT-PCR amplification was carried out in a 96 Light-Cycler machine (Roche Applied Science). All reactions were carried out in 20 μl volumes that comprised of 10 μl Fast SYBR green master mix (2×), 2 μl reverse primer, 2 μl forward primers, 4 μl dH_2_O plus 2 μl cDNA template. PCR was initiated by denaturation at 95 °C for 10 min followed by 40 cycles at 95 °C for 3 s, 60 °C at 30 s and 72 °C for 30 s amplification. Melting curve analysis for each amplicon was done to determine the amplification specificity while gel electrophoresis was used for verification of each single product. Transcription levels for each target gene were quantified relative to the internal control housekeeping β-actin gene using the delta-delta method^42,43^.

**Table 1:** Primers used for gene expression analysis.

Gene	Primer sequence		Genebank Acc. No	Bp Size	Reference
TLR3	F	TGCAACACTCCACTGACTTAC	ACC# JN589795.1	99	This study
	F	GCTCAGTATGTAAAGAGCCTGAA			
TLR7	F	TCAGCAGGGTGAGAGCATAC	XM_00547 7981.1	143	^46^
	R	ACATATCCCAGCCGTAGAGG			
β-actin	F	GATCTGGCATCACACCTTCTAC	ACC# KJ126772	104	This study
	R	TCTTCTCCCTGTTGGCTTTG			
Mx	F	GGATCCTGATGGAGAGAGGA	XM_003460517.2	136	^47^
	R	GCATTTGACCACCATGTAGC			
IgM	F	AGGAGACAGGACTGGAATGCACAA	KJ676389.1	117	^48^
	R	GGAGGCAGTATAGGTATCATCCTC			
IFNβ	F	TGGTCTGATTGTCGTCCTGT	XM_003452995.1		^47^
	R	CGCTCCATGTCTCTGTCAGT			
TiLV Seg 3	F	TCCAGATCACCCTTCCTACTT	KU751816	109	This study
	R	ATCCCAAGCAATCGGCTAAT			

Virus quantification was carried out using cDNA synthesized from brain, liver, headkidney and spleen samples using primers targeting TiLV segment 3 (KU751816) (Table 1) by generating a linear standard curve using supernatants from TiLV infected TFC#10 cells where the concentration varied from 10^6^ TCID_50_/mL to 10^0^ TCID_50_/mL.

### Histopathological examination

Tissues were fixed in 10% phosphate-buffered formalin and histopathological examination was carried out after embedding fixed tissues in paraffin blocks followed by microtome (Microm HM3555, Microm International GmbH, Walldorf, Germany) sectioning and staining using hematoxylin and eosin according to standard methods. Stained slides were examined under a light microscope for histopathological changes and images were captured using a Zeiss Axioscope equipped with a Leica SFC 420 camera.

### Data analysis

Data generated from RT-PCR gene expression was entered into Microsoft Excel sheets and exported to GraphPad Prism version 8.0.0 for Mac, GraphPad Software, San Diego, California USA, or Stata 15, StataCorp, 4905 Lakeway Drive, College Station, Texas 77845 USA, for statistical analysis and image preparation. Ct values from gene expression were used to estimate relative expression of the genes with β-actin as the internal housekeeping control gene^43^ and expression was presented as −∆∆Ct values. Variation in the expression levels was analyzed using either a one-way ANOVA in GraphPad prism8 or Stata15 or a non-parametric test (Kruskal Wallis) when residuals were found non-conformant with a normal distribution. Significance level was set at 5% (p < 0.05). Where relevant a regression analysis was also performed followed by testing for normality distribution of residuals. The different segments of TiLV (KU751814-KU751823) were used as basis for assessment of nuclear and nucleolar localization regions using online resources (cNLS mapper)^44,45^.

## Supplementary information

Supplementary file1
