# Supplementary material for: Tilapia lake virus downplays innate immune responses during early stage of infection in Nile tilapia (Oreochromis niloticus)
Source: Sci Rep. 2020 Nov 23;10:20364. doi: 10.1038/s41598-020-73781-y (PMC7684318; doi:10.1038/s41598-020-73781-y)
Supplement: Supplementary file 1 — Supplementary file1 [file 41598_2020_73781_MOESM1_ESM.pdf]

Tilapia lake virus downplays innate immune responses during early stage of infection in Nile tilapia (*Oreochromis niloticus*)

Kizito K Mugimba[1,2], Mustapha Lamkhannat[1], Saurabh Dubey[1], Stephen Mutoloki[1], Hetron M. Munang'andu[1] and Øystein Evensen[1]

1- Norwegian University of Life Sciences, Faculty of Veterinary Medicine, Department of Basic Sciences and Aquatic Medicine, PO Box 369, 0102 Oslo, Norway

2- Makerere University, College of Veterinary Medicine Animal resources and Biosecurity, Department of Biotechnical and Diagnostic Sciences. P.O Box 7062, Kampala, Uganda.

Correspondence: oystein.evensen@nmbu.no

Supplementary Figures 1 - 4.

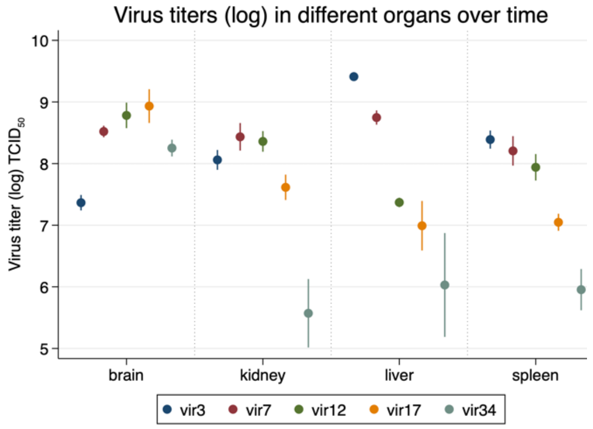

Supplementary Fig. 1. Virus titers in different organs over the course of the challenge period.

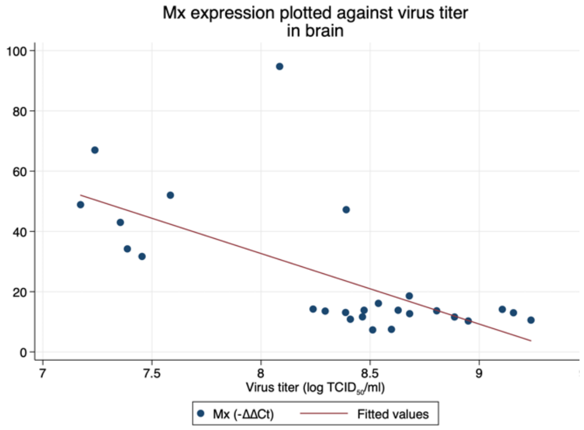

Supplementary Fig. 2. Mx expression plotted against virus titer.

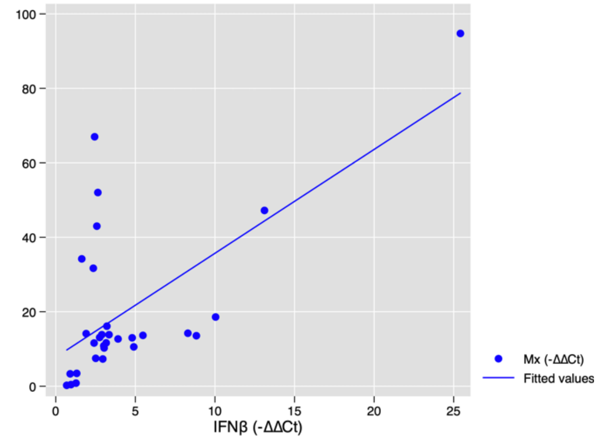

Supplementary Fig. 3. IFN-β plotted against Mx for the brain over the course of the challenge period, r^2=0.65.

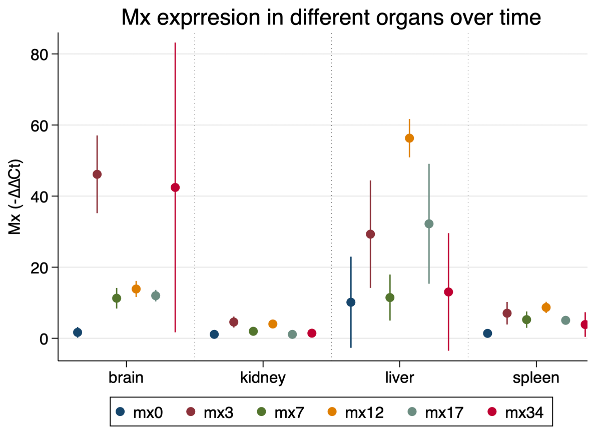

Supplementary Fig. 4. Mx expression in different organs over the course of the challenge period. Expression is significantly higher in brain compared to kidney and spleen at all timepoints, $p \leq 0.025$.
